# Killing Kira, Letting Tom Go?—An Empirical Study on Intuitions Regarding End-of-Life Decisions in Companion Animals and Humans

**DOI:** 10.3390/ani12192494

**Published:** 2022-09-20

**Authors:** Kirsten Persson, Felicitas Selter, Peter Kunzmann, Gerald Neitzke

**Affiliations:** 1 Applied Ethics in Veterinary Medicine Group, Institute for Animal Hygiene, Animal Welfare and Farm Animal Behaviour, University of Veterinary Medicine Hannover Foundation, Bischofsholer Damm 15, Geb. 116, 30173 Hanover, Germany; 2 Institute for Ethics, History and Philosophy of Medicine, Hannover Medical School, Carl-Neuberg-Str. 1, 30625 Hanover, Germany

**Keywords:** veterinary ethics, end-of-life decisions, medical ethics, quantitative research

## Abstract

**Simple Summary:**

End-of-life decisions present challenges in both human and veterinary medicine. Legally, the options are clearly defined in the respective fields. The legal differences, however, are not necessarily mirrored in personal judgements by different stakeholder groups involved in the decision-making processes. In this study, veterinary and medical professionals as well as a control group of laypersons were asked to choose treatment options in six different fictional end-of-life scenarios for human and for animal patients, differing in age, gender, and, in case of the human patients, in terms of their state of consciousness. Interesting differences and congruencies—within and between stakeholders but also animal and human patients—can be found in the results, especially with regard to the reasons study participants gave for their choices.

**Abstract:**

Veterinary and human medicine share the challenges of end-of-life decisions. While there are legal and practical differences, there might be parallels and convergences regarding decision-making criteria and reasoning patterns in the two disciplines. In this online survey, six variants of a fictitious thought experiment aimed at pointing out crucial criteria relevant for decision-making within and across both professional fields. The six variants introduced four human and two animal patients with the same disease but differing in age, gender and, in case of the human patients, in terms of their state of consciousness. Participants could choose between four different treatment options: euthanasia, continuous sedation, a potentially curative treatment with severe side effects and no intervention. Study participants were human and veterinary medical professionals and an additional control group of lay people. Decisions and justifications for the six variants differed but the three groups of participants answered rather homogeneously. Besides the patient’s “suffering” as a main criterion, “age”, “autonomy” and, to a lesser extent, “species” were identified as important criteria for decision-making in all three groups. The unexpected convergences as well as subtle differences in argumentation patterns give rise to more in-depth research in this cross-disciplinary field.

## 1. Introduction

Kira is sick. She suffers from a serious lung disease that will eventually make her suffocate if not treated. Her doctors inform you about her options: They could administer a tablet A that will end her life immediately and painlessly. They could give Kira a different tablet B that would sedate her so that she would fall asleep and die painlessly from her disease within the next two weeks. Tablet C has a 70% chance of curing Kira. However, she would suffer from the side effects of that treatment for a period of 6 months, including nausea, vomiting, and pain. What do you consider the best option for Kira?

It depends, you might answer. It depends on several context-specific aspects we did not give you here. Our study focused on some of those very aspects that might make a difference for people’s decisions and explanations. Who is Kira? Does it, for example, matter, if Kira is young or old? Would it be different if Kira was Tom? Is it relevant for your decision to which extent Kira is autonomous? And does your decision depend on whether Kira is a human patient or, let’s say, a dog? Besides, it might be important who you are. Are you someone who professionally decides on therapy limitations or companion animal euthanasia on a regular basis? Or are you any other citizen who might have been personally involved in care for someone in their final days of life?

In this study, we want to tackle the vibrant discourse on ethical aspects in end-of-life decisions (EOLD), empirically and from an unconventional angle. We are interested in convergences and divergences regarding the ethics of EOLD in human and companion animal medicine. Legally, the framework for decision-making differs substantially between human and veterinary practice and, besides, from country to country. A recent court ruling of the German Federal Constitutional Court initiated a new debate on physician-assisted suicide in Germany [1] which had been legally restricted since 2015 by the German Criminal Code. At the same time, the German Animal Welfare Act protects animal life in a unique way. Paragraph 1 declares human responsibility for life and welfare of animals and prohibits causing pain, suffering, or harm to animals without reasonable cause.

Within this framework, physicians as well as veterinarians counsel patients, patient owners and family members in EOLD and decide on therapy options. In veterinary practice, euthanasia is an established and fundamentally unquestioned means to end a patient’s suffering if other options are ruled out, or if it is impossible to administer the adequate therapy to the patient [2]. Human physicians are provided with a different toolbox in EOLD. Individual pain therapy, sedation but also spiritual counselling frame the final phase in palliative and hospice care. However, recent research findings suggest parallels or even convergences between EOLD in veterinary and human medical practice, such as the role of relatives, the importance of the individual patient’s preferences, challenges in communication, preferred circumstances of the final phase [3] and death ideals [4]. Habituated to legal constraints and common practice, human and veterinary medical professionals may not be used to challenging their own thoughts on their decision-making criteria [5], and much less on those of the respective other profession. 

In this study, we wanted to shed light on precisely these decision-making criteria in EOLD in veterinary and human medical practice and also in comparison to the public perspective on this matter. Therefore, we designed the thought experiment briefly introduced above with six variations concerning patient properties, including the patient being a dog or a human, being young or old (infant, child, elderly patient), female or male and, in case of the human patient, one variation with the patient being in a coma. In offering four treatment options—no treatment, euthanasia, continuous sedation and a potentially healing treatment with serious side effects—we mixed alternatives that are actually available in human and/or veterinary medicine and thereby took away the legal and practical restrictions that exist in real life. We recruited participants from the veterinary and human medical field and added a control group without a background in (veterinary) medicine. By parallelizing the cases of human and nonhuman patients and ascribing the same disease, we wanted to explore those characteristics that make the cases different for our participants and identify potential thresholds in the assessment of these situations. In addition to their choices, we asked for their criteria and explanations.

Depending on the factors specified in our thought experiment, we expected to find differences regarding:

(1) The species of the patient (dog, human). Despite the explicit hint that all options are legally and technically possible in our hypothetical scenario, we expected participants to differentiate between an animal and a human patient in EOLD. That difference can be expected in all three groups, assuming the fundamental human habit of differentiating between ending animal and ending human life. More specifically, though, one research question would be to determine whether all three groups perceive this difference as equally important and whether the (pattern of) reasons participants mention for their decisions is similar between the three groups.
(2) The patient’s age. There are—at least—three separate dimensions of the factor “age” that may come into play in EOLD. First, an elderly patient might not be able to compensate for certain side effects as well as a younger one. She might suffer more, take longer to recover, or have disadvantages due to comorbidities. Participants might not want to put an elderly patient though the suffering (from the side effects of the treatment) and therefore would rather let them go (from here: the “putting-through dimension”).

The second dimension is a calculation of a measure’s effectiveness, known from medical ethics as “QALY”, quality-adjusted life years [6]. The main question is: How much quality-time of life can be obtained when investing or sacrificing something, in this case half a year of pain and suffering from the side effects of the treatment? Participants taking into account this calculation need, first of all, information on the remaining life-expectancy of the respective patient. The degree of knowledge on our dog-patient’s life expectancy might differ between veterinary and human professionals (and the control group) depending on how well they are generally informed about dogs, whereas knowledge of human life expectancy is expected to be distributed equally among our participants. Furthermore, they need information about the chances of recovery, which are provided in the scenario’s text. That way they can elaborate on their calculation process (from here: “the QALY-dimension”).

A third argument referring to age is known as “fair innings”, which has recently gained attention again when deliberating decision-making during a pandemic. The basic idea “reflects the feeling that everyone is entitled to some ‘normal’ span of health (…) and anyone failing to achieve this has been cheated, whilst anyone getting more than this is ‘living on borrowed time’” [7] (p.117). This principle is mainly applied when allocating scarce resources, which is not an issue in the presented study. Nevertheless, the idea that someone has already lived their full live span might provoke the thought that there is no need to expose them to a risky and painful procedure for some additional lifetime whereas those who have not yet lived that long should get the chance at any “cost” (from here: the “fair-innings-dimension”).

To what extent all of these arguments are presented for animals and humans and used by all groups is the main research question regarding “age”.

(3) The patient’s autonomy. The reason for including an infant and a person in a coma was to point towards the so-called argument from marginal cases. While there are differences between most humans and most nonhuman animals, there are cases of certain humans—the “marginal” cases—who share morally relevant properties with certain nonhuman animals. This group consists, e.g., of infants and persons with dementia or other mental impairments. Like animals, they are not autonomous, meaning that they are not able to tell us their preferences and we are not able to explain future benefits from current treatments or actions to them. At the same time, they are able to suffer and clearly express suffering. In the study, the human patient in a coma, additionally, is never expected to gain autonomy again whereas the infant bears the potential to become an autonomous person later in her life and the 11-year old already is autonomous to a certain degree but still dependent on the decision-making of adults.

Besides the debate on animal autonomy, there is the much less controversial attitude that experienced humans (like companion animal “owners” or veterinarians) are able to access (companion) animal preferences. Therefore, participants might be interested in the question as to which extent the animal patient in the given scenario expresses their preference to continue living or rather to end their life.

## 2. Materials and Methods

### 2.1. The Thought Experiment

The fictitious scenario was designed by the authors and pilot-tested among colleagues (with a background in biomedical ethics, human and veterinary medicine and the humanities). According to their feedback, we adjusted wording and clarified ambiguities. The scenario reads as follows (translation from German, original version in the Supplementary Materials S1):

Tom is an 11-year-old male Labrador. He has been diagnosed with a progressive lung disease and his current condition is poor. He has breathing difficulties, no longer eats and has little interest in everyday activities such as walks. The options are as follows:

- If no medication is given, Tom will suffer from shortness of breath and suffocate in a short time.
- If tablet A is administered, Tom will immediately fall asleep without pain or fear and die.
- If tablet B is given, Tom will be sedated so that he does not feel fear, pain or discomfort. He is expected to die from his illness within the next two weeks.
- If tablet C is given, Tom will experience severe side effects such as nausea, loss of appetite, dizziness, severe fatigue and listlessness for about six months. After that, there is a 70% chance that his lung will be cured. There is a 30% chance that lung failure cannot be stopped in this way.

### 2.2. Which Option Do You Consider to Be Right?

The participant selected from a list: Tablet A (from here “euthanasia tablet”), Tablet B (from here “sedation tablet”), Tablet C (from here “curative tablet”) or no therapy. Afterwards, they were asked to explain their decision in a text box: “I made this choice because…”

Wording was the same for a 70-year-old human, an 11-year-old human, and a 2-year-old Labrador Retriever. Wording differed for a 30-year-old human in an irreversible coma for tablet C because a coma patient is not expected to suffer from the listed side effects:

- If tablet C is given, there is a 70% chance that his lung will be cured after 6 months. Tom will not feel the usual side effects such as nausea, dizziness and severe fatigue. There is a 30% chance that lung failure cannot be stopped this way.

Additionally, we adapted the description of the current state of the 6-month-old infant to make it plausible. The sentence “He has breathing difficulties, no longer eats and has little interest in everyday activities such as walks.” was replaced by “He has breathing difficulties, no longer drinks and has little interest in contact with his environment.”

Half of the participants were assigned the questionnaire with the six different patients in this scenario called Tom. The other half got the same scenarios but with a female patient named Kira. The scenarios were displayed in randomized order to avoid framing effects.

### 2.3. Recruitment and Data Collection

As representative of the ethics committee, the animal welfare officer of the Veterinary University Hannover approved the study in March 2020. Recruitment for the online survey took place between May and August 2021. We used social media groups (closed Facebook groups for human medical and veterinary medical professionals) and email distribution lists for employees in human and veterinary hospitals to recruit participants from the three different groups. For the lay public group, we asked them to forward the emails and announcements in the social media groups to further acquaintances, neighbors, family members, etc. The aim was to reach at least 100 participants of each group (human medical and veterinary professionals, lay public). LimeSurvey (LimeSurvey GmbH, Hamburg, Germany, www.limesurvey.org, last accessed 7 January 2022) was used as a survey tool. The survey was started 6 May and finished on 25 August 2021 when a sufficient number of responses had been received.

### 2.4. Analysis

For the quantitative analysis and creation of the diagrams, we used Microsoft^®^ Excel (Version 2016) and IBM SPSS Statistics Professional. Quantitative analysis was performed by the first author and discussed with the other authors. The results were analyzed descriptively and checked for between-group differences among the answers for each scenario variant with a Chi-squared test. For the analysis of the open-ended responses, MAXQDA (2020, VERBI Software, Berlin, Germany) was used to structure answering patterns and to obtain an overview regarding emerging themes and their frequencies. As the free texts were very short (often only one word, sometimes a few words and occasionally up to two sentences.) it would not have been meaningful to conduct a thorough content analysis of qualitative research. However, explanation patterns were identified by going through the answers iteratively and ascribing codes in MAXQDA. The dominant explanations were expected to follow the study design, as the six vignettes differed most obviously regarding species of the patient, age and autonomy. However, the coding was done inductively and no codes or themes had been defined in advance.

## 3. Results

### 3.1. Demographics

Of 1096 participants who started the survey, 673 completed it (61%). Of those, 98 identified as human medical professionals and an additional 15 participants were added to this group based on their information regarding their profession (e.g., nurses, paramedics, former physicians, hospice volunteers). This decision was based on the most relevant criterion of being potentially involved in human end-of-life situations beyond private experience. In total, 17% (*n* = 113) of the participants were assigned to the group human medical professionals, from here “HumMed” group. 230 respondents identified as veterinarians and after adding animal caretakers (zoo), farmers, a lab worker, an animal physiotherapist and alternative animal health practitioners to the group, 36% (*n* = 244) formed the group of animal professionals, from here “VetMed” group. Again, the decision was made because those people potentially work with animals in end-of-life situations. The remaining 47% (*n* = 316) were the control group participants.

### 3.2. Quantitative Results

**Figure 1:**
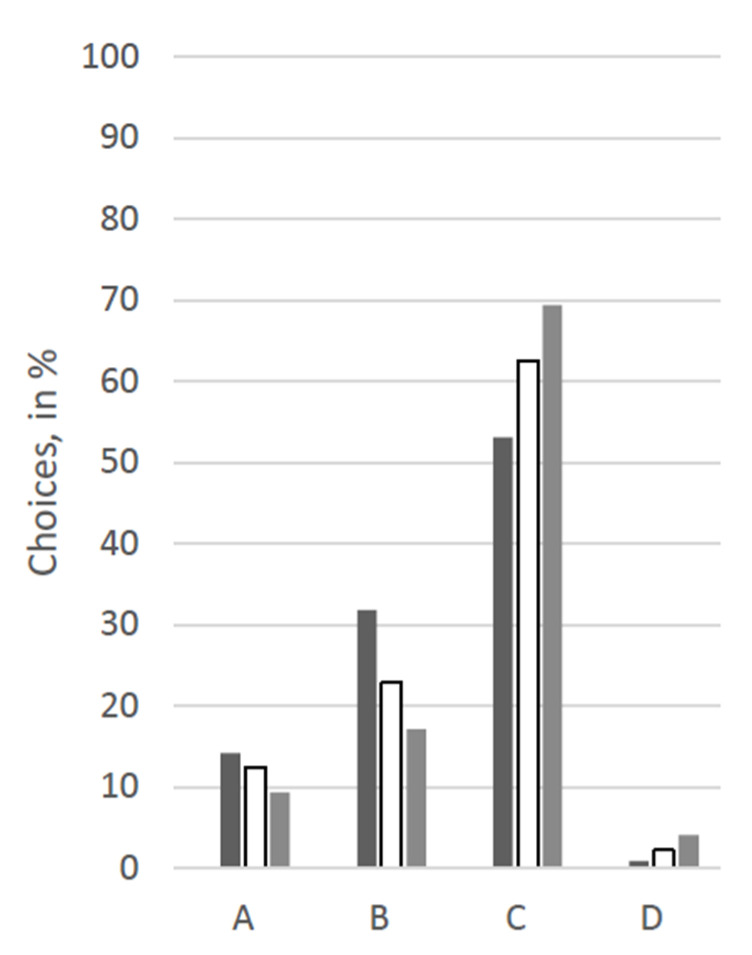
Answers for the scenario “70-year-old human” in %. Legend: black: human medicine group; white: control group; grey: veterinary medicine group; A: Euthanasia tablet, B: Sedation tablet, C: Potentially curative tablet, D: No treatment.

**Figure 2:**
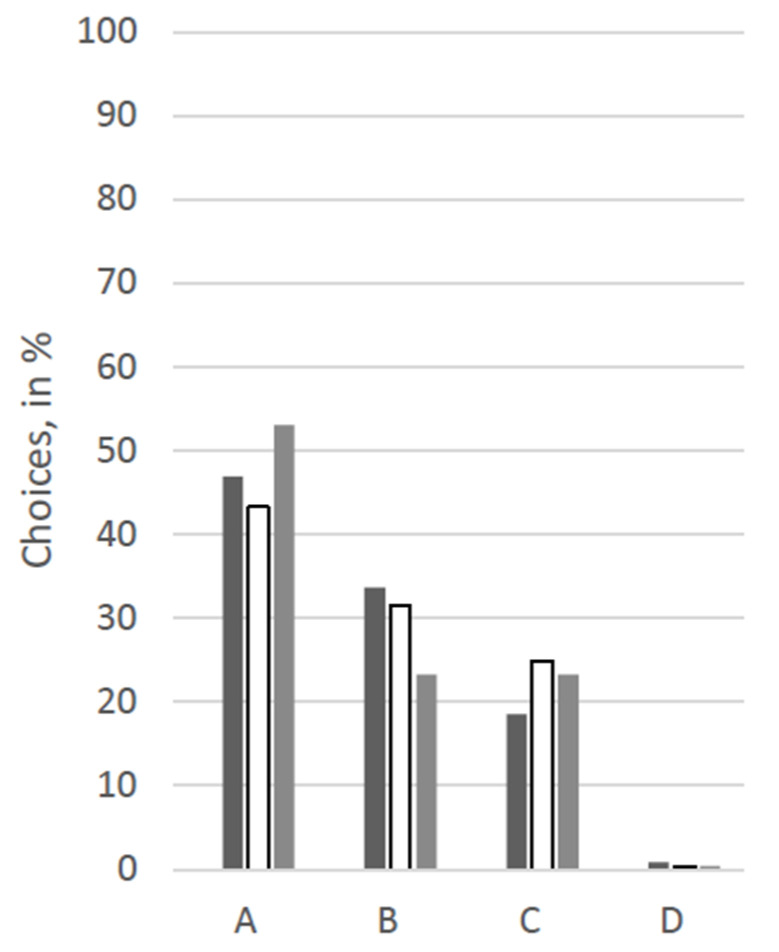
Answers for the scenario “11-year-old dog” in %. Legend: black: human medicine group; white: control group; grey: veterinary medicine group; A: Euthanasia tablet, B: Sedation tablet, C: Potentially curative tablet, D: No treatment.

**Figure 3:**
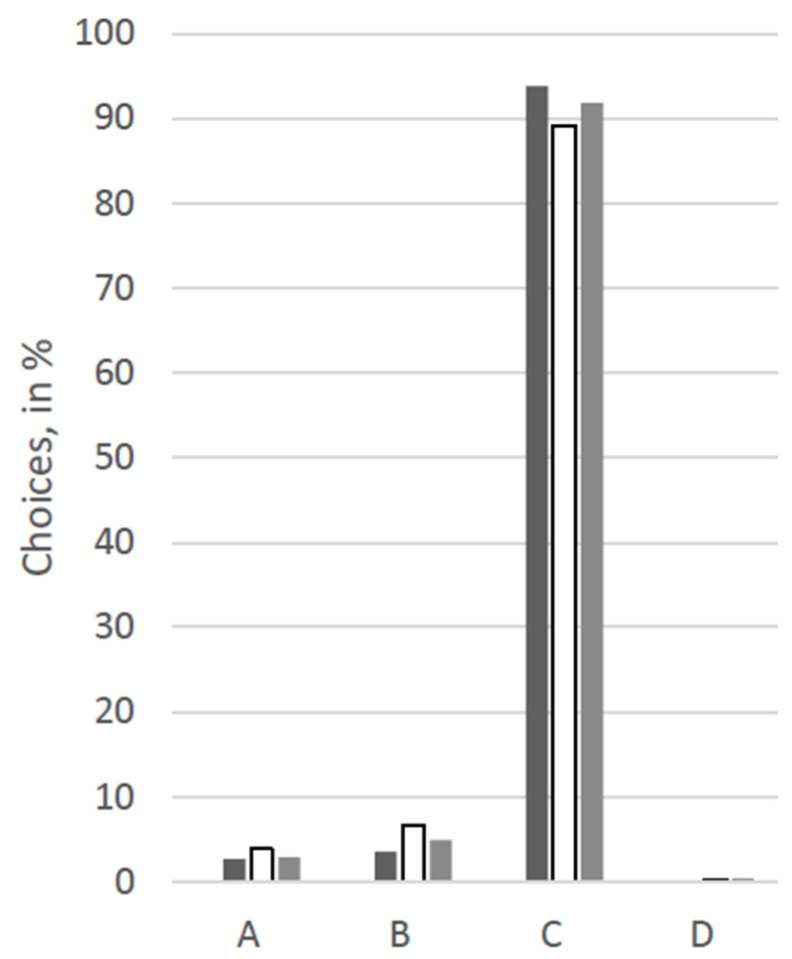
Answers for the scenario “11-year-old human” in %. Legend: black: human medicine group; white: control group; grey: veterinary medicine group; A: Euthanasia tablet, B: Sedation tablet, C: Potentially curative tablet, D: No treatment.

**Figure 4:**
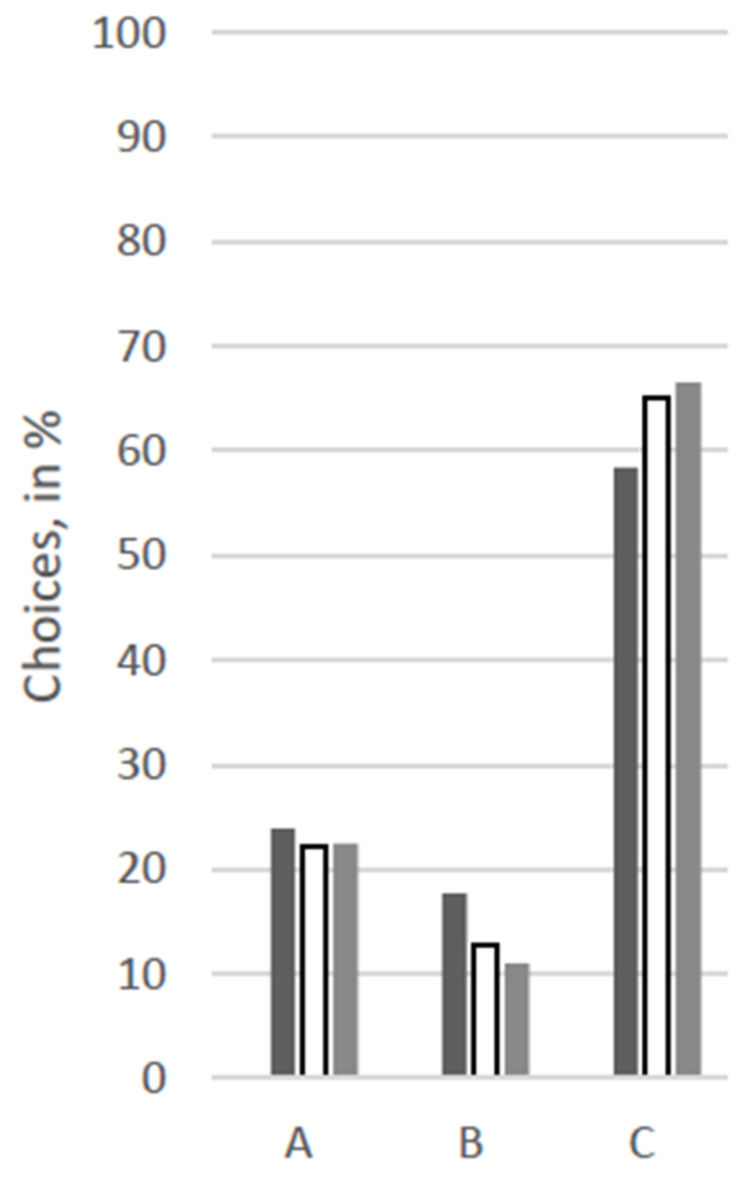
Answers for the scenario “2-year-old dog” in %. No participant opted for “D” in this scenario. Legend: black: human medicine group; white: control group; grey: veterinary medicine group; A: Euthanasia tablet, B: Sedation tablet, C: Potentially curative tablet, D: No treatment.

**Figure 5:**
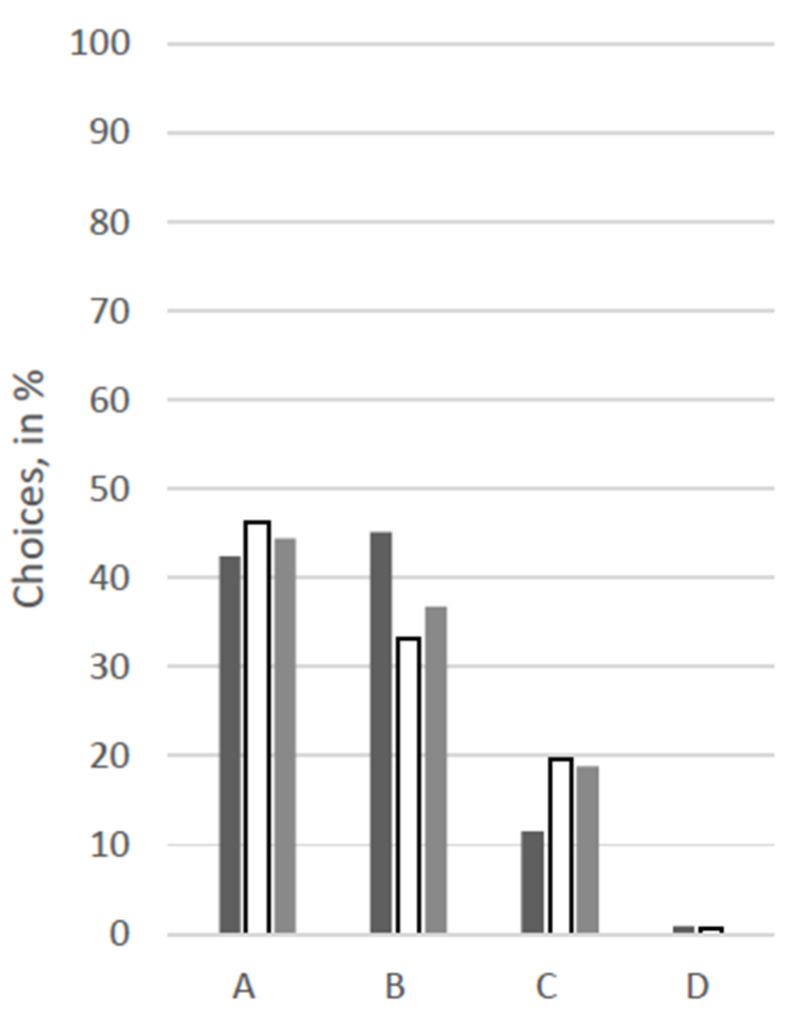
Answers for the scenario “30-year-old human in a coma” in %. Legend: black: human medicine group; white: control group; grey: veterinary medicine group; A: Euthanasia tablet, B: Sedation tablet, C: Potentially curative tablet, D: No treatment.

**Figure 6:**
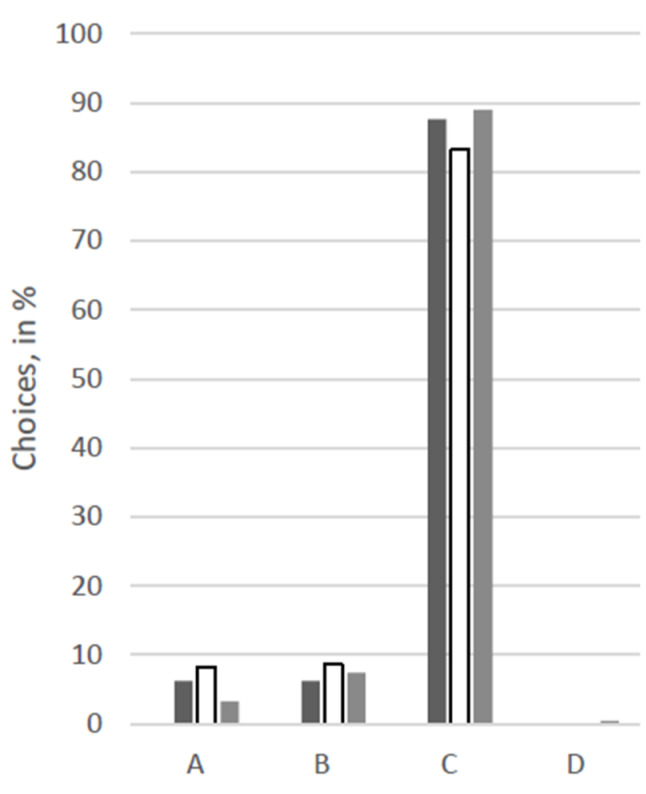
Answers from the scenario “6-months-old human” in %. Legend: black: human medicine group; white: control group; grey: veterinary medicine group; A: Euthanasia tablet, B: Sedation tablet, C: Potentially curative tablet, D: No treatment.

For an overview of the answers regarding choices for therapy options, see Figure 1, Figure 2, Figure 3, Figure 4, Figure 5 and Figure 6. 

At first glance, response patterns differed substantially between the different patients, i.e., between the scenarios, but not so much between the groups of participants (see Figure 1, Figure 2, Figure 3, Figure 4, Figure 5 and Figure 6). For both the 11-year old and the 6-month-old human patient, more than 80% of all groups opted for the curative treatment (C) that gave the patient a chance of surviving the disease at the price of severe side effects. More than 50% of all participants chose this option for the 2-year-old dog and the 70-year-old human. For the human coma patient and the 11-year-old dog, most participants voted for the euthanasia tablet (A) (>42% and >47%) or the sedation tablet (B) (>37% and >23%). In all scenarios, option D “no therapy” was hardly ever chosen (0–4%). 

Significant between-group differences (*p* < 0.05) occurred in a Chi-squared test for the 11-year-old dog, in which case more than 53% of the VetMed group voted for the euthanasia tablet and ca. 23% each for sedation tablet and the curative tablet. The other groups chose the euthanasia tablet less frequently (Hum: 47%, Contr: 43%) and the sedation tablet more frequently (Hum: 34%, Contr: 31%). Similarly, in the scenario with the 70-year-old human patient, the HumMed and VetMed group answered significantly differently (*p* < 0. 05). While almost 70% of the VetMed group opted for the curative treatment (9% euthanasia, 10% sedation), only 53% of the HumMed group chose that option (14% euthanasia, 32% sedation). A third significant difference (*p* > 0.05) is displayed in the coma patient scenario, in which case human professionals opted for the sedation more often (45%) than the other two groups (Contr: 33%, VetMed: 37%) and for the curative tablet less frequently (Hum: 11%, Contr: 20%, VetMed: 19%). 

### 3.3. Free-Text Explanations

The free-text boxes offered participants the opportunity to explain their choices. They allowed for research questions regarding the participants’ reasons, particularly as the two professional groups voted rather similarly for most scenarios. Reasons and arguments behind the (hypothetical) decisions might help to identify the categories (species, age, state of mind, etc.) which guided and motivated the decisions made. The texts varied in length and explanatory strength. Some participants only wrote one word picking up the demarcating fact of the specific scenario such as “young” or “coma”. Some explicitly wrote that they had been undecided considering two or more options. Others explained why they excluded some options and, consequently, voted for the remaining one. Additionally, participants referred to personal wishes and experiences. 

Before looking into the response patterns relating to the three research foci presented in the introduction, the authors give a brief overview of the most common explanations and reasons. It is worth noting that the most common reasons or justifications for choosing one of the four options were comparable throughout the scenarios and the three participant groups.

Overall, most participants explained their decision for euthanasia tablet A or sedation tablet B by reduction of suffering for the patient, no matter if dog or human. Occasionally, participants specified “suffering” in terms of the potential side effects of the treatment (tablet C) or in terms of the pain and the imminent suffocation due to the lung disease. At the same time, most of them did not explain why they did not opt for one of the other two tablets, which would have also reduced suffering. If they did, they commonly mentioned the chance to say goodbye for a couple of days, which is implied in continuous sedation. For both options and both groups of patients, several participants stated that this was a good, pleasant or dignified way to die (“A peaceful redemption seems to me the most dignified”, Scenario: 70-year-old human, group: VetMed, decision B; in short: human, 70y, VetMed, B). Only VetMeds call option A “euthanasia” or “putting to sleep”. Complementary to this, human medical professionals talk about a “clear palliative situation” (coma patient scenario), and that “B is not active assisted dying but enables dying without suffering” (coma patient scenario) when administering sedation tablet B.

Some HumMed participants choosing tablet A had different explanations, besides the main reasons, i.e., that there is no or too little chance of cure and too much suffering for the patient. For the coma patient, they state that, in their eyes, “it is undignified to delay death any longer”, “an irreversible coma is not a state worth living in—therefore, in the case of additional severe suffering, active assisted dying is also justified”, “life is so incredibly precious, but not at any cost” and, taking family members into consideration, “perhaps suffering and burden can be lifted from the family”.

The vast majority of those who decided on curative tablet C explained their choice by good healing chances (70%).

Occasionally, participants did not trust the description of the scenarios, called them unrealistic or medically wrong, or interpreted additional circumstances, such as this animal professional, choosing tablet A for the coma patient: “Tablet B will probably sedate Kira and take away her stress, but in the end she will probably suffocate, which is a cruel death in my eyes. Therefore, I have decided to use tablet A, which will relieve Kira of her suffering without any stress or pain”, or this human medical professional explaining why they chose sedation tablet B for the coma patient: “Hmm, actually, something like that (i.e., the described situation) is more likely to be ended by withdrawing from therapy and not by giving a tablet (…)”.

The authors’ research focus was on the factors laid out in the introduction:

(1) Species: To what extent do explanations refer to the patient’s species?

Despite species being an obvious difference between the patients in the scenarios, it was not a factor the participants frequently explicitly mentioned in their reasoning. Rather, participants referred to those animal properties they do not share with (most) human patients: nonhuman animals do not understand the purpose of a long-lasting treatment; they are not able to tell us their preferences; we might not be able to estimate the extent of their suffering, etc. Those criteria, however, were also true for the 6-month-old or the comatose human patient, which is why, at least for the infant, participants argued similarly when explaining their decisions (see aspect 3. “Autonomy”). 

Particularly in the answers of the control group to the animal patient scenarios, there are a few explicit references regarding differences between nonhuman animals (or, more specifically, dogs) and humans. From the short statement that “he is a dog” (dog, 11y, Control, A) or, similarly, the insight that “he is 11 years old and an animal is not a human” (dog, 11y, Control, A) to the more elaborate view that “half a year with side effects is too long for an animal” (dog, 11y, Control, A), participants offered justifications for immediately putting the animal to sleep. Human medical professionals referred to the animal’s lack of ability to say goodbye (dog, 2y, HumMed, A) or the inability to communicate suffering (dog, 2y, HumMed, A). Some participants of the animal group, in contrast, presented the minority opinion that the treatment tablet would be the right choice as “animals don’t ask whether something is ‘worth it’, they accept things as they are. That’s why he survives the half year” (dog, 2y, VetMed, C), or, emphasising the emotional state of the patient’s family, “you also don’t suffer as much regarding the side effects as you do with a child” (dog, 2y, VetMed, C). Apparently struggling with a decision, an animal group participant choosing C admits that “6 months is an eternity even for a 2-year-old dog, but I think I would want to give it a try”.

Participants mention the species in the free-text answers to the human patient scenarios even less frequently. One VetMed participant states that there is a “small difference between humans and animals. Euthanasia critical issue” when arguing for their decision to administer sedation tablet B to the 70-year-old patient. 

Strikingly, a HumMed participant opting for tablet A answers that the 6-month-old human is “too young to actively say goodbye”, implicitly referring to a parallel with animal patients. The idea that “every human has the right to a dignified and painless death”, (e.g., human, 70y, Control, B) is not mentioned for the animal scenarios.

(2) Age: Do the participants elaborate their understanding of the relevance of age (putting-through-dimension, QUALY-dimension, fair-innings-dimension)?

Age is one of the most-mentioned reasons in the free text answers for the elderly, but also for the explicitly young patients. Besides vague statements like “because of the age” or “because she is old,” participants elaborate in several ways to what extent the factor is relevant for their decision-making. The statements suggest that all three dimensions of age come into play in the participants’ reasoning:

Putting-Through Dimension

Participants argued from both angles: for choosing treatment tablet C, such as “a young body can take more” (dog, 2y, HumMed, C; human, 11y, Control, C), or for letting the patient go, because “Kira is already elderly, I wouldn’t want to put her through the strain” (human, 70y, Control, B). The wording was often similar for human and animal patients: “Since the dog has reached a fairly advanced age of 11 years, you don’t want to put the old guy through a torturous therapy but rather let him ‘go’ peacefully” (dog, 11y, HumMed, B). Some participants explicitly compared the two different ages concluding that “here the decision is different, because at 2 years old (…) the body might be able to cope better” (dog, 2y, Control, C).

QALY Dimension

The basic idea that the quality time gained should be weighed against the duration of the suffering while undergoing the treatment was brought up frequently. However, the argument was used for different decision outcomes, depending on (a) the knowledge regarding life expectancy and, (b) the judgement regarding how much potential good-quality time would be worth the sacrifice of—at least—half a year of bad-quality time. 

The knowledge regarding life expectancy of dogs in general and Labrador Retrievers in particular was rather heterogeneous among the groups of participants according to the estimations within the free-text answers. In the control group, 14, 15 or 16 years were mentioned, whereas the VetMed group brought up the specific life expectancy quite frequently between 10 and 14, mostly 12 years. Consequently, those participants evaluated the question of the ratio between the expected life years with a good quality and the comparably short time of suffering differently for the elderly dog:

- “I have no idea whether 11 years is a high age in dogs. If it is and her lifetime is almost at the end anyway, I would have chosen A.” (Control, C)
- “Despite the high age, there is a high chance of recovery after 6 months. However, tablet A would also be justified.” (VetMed, C)
- “70% survival rate worth the side effects.” (HumMed, C)
- “Labrador is at the target age (10–12 years), no further problems mentioned. Tablet C offers only 0% quality time and 70% quality time for half of the time in the last year of life, but on average only a 35% chance—in other words, a little more than 2 months. This is better than B with 2 weeks.” (Control, C) (This is meant as an example for a participant using a weighing calculation to explain their decision, despite the calculation process being hard to follow/flawed.)

However, there were participants who seemed to judge the 6 months period with severe side effects to be too much for an animal per se, because “proportionally, the animal suffers for an unnecessarily long time” (dog, 2y, HumMed, A).

In contrast to that: “The 70% chance of recovery justifies the 6 months of side effects for me, as the animal is at the beginning of its life and could easily live another 10 years” (dog, 2y, VetMed, C). Another veterinarian states that, “we treat diseases with a worse prognosis!” (dog, 2y, VetMed, C).

Fair Innings Dimension

A VetMed group participant admitted: “I am struggling a lot with my answer, as age should not be a reason that influences the decision” (dog, 2y, VetMed C). Nevertheless, the perspective that the elderly patient, be it human or dog, has already lived a complete life came up in many statements, especially when opting for the sedation tablet B:

- “Tom has already had a relatively long life (…). Through Tablet B, Tom can spend his last weeks peacefully and with dignity, have his family around him one last time and everyone can say goodbye and prepare for the inevitable.” (human, 70y, Control, B)
- “The good man has lived his life.” (human, 70y, Control, B)
- “Life expectancy at the end.” (human, 70y, VetMed, B; dog, 11y, HumMed, A etc.)
- “11 years is a noble age for a (usually very agile) Labrador. But I wouldn’t want him to suffer.” (dog, 11y, HumMed, B)

The general problem of age comparability in humans and companion animals was at least touched on in passing, e.g., when a HumMed participant elaborated regarding the 70-year-old human patient: “It’s difficult, his general condition would be interesting. Age 70 is theoretically no longer as old as it used to be”; or when a VetMed participant voting for B for the 11-year-old dog states: “Half a year with the side effects mentioned is a very long time in relation to the average life expectancy of the Labrador (this period would be several years in the example with the 70-year-old woman).”

(3) Autonomy: To what extent is it relevant for the participants’ choices if the patient is, has been, or will be autonomous?

The most relevant explanations with regard to autonomy belong to the answers in the scenarios with non-autonomous patients. On the one hand, for the infant, several participants explicitly or at least implicitly emphasize commonalities between non-autonomous animals and humans:

- “Because for me a baby can as little communicate as an animal and I don’t want to inflict unnecessary pain.” (human, 6 months, HumMed, A)
- “The baby doesn’t understand what’s happening to him” (human, 6 months, Control, A) (an argument that was also often brought up for not choosing the therapeutic tablet for an animal patient.)
- “Probably, I’ll get in trouble now because I let everyone die, but for me this would be the same as for the dog.” (human, 6 months, VetMed, A)

On the other hand, HumMeds opting for A or B in the coma patients hardly ever refer to the fact that the patient is not/no longer autonomous. Only one participant asks if there was an advance directive, giving credit to the patient’s choice. One control group participant explains their choice for “B” with an unusual ascription of autonomy, though: “(…) If the body cannot do it without outside help, one should not prolong the suffering, but give the soul the opportunity to choose the time—free of pain—itself.”

The control group members opting for A or B in the coma patient rather refer to their own experiences with death/dying (“I have experienced a similar case. The patient herself did not suffer. For the relatives it was a relief to have come to this decision after months of struggle, incidentally supported by the patient’s family doctor who had been looking after her for many years”), or to their own preferences (“If it was me, I would want it that way”), besides the most commonly mentioned factors that the patient is in a coma, suffering and the chances of cure are too low. One participant excludes the other options: “Has no desire (for) B, and no benefit from C”. Another one adds “if it was allowed” when choosing A, showing their awareness of the legal situation regarding killing on demand. Several participants refer to a potential advance directive and a few mention the option to donate organs as a reason for choosing A.

Among the veterinarians, too, we find an answer explicitly including the patient’s preferences: “For me, an irreversible coma is already sufficient reason for euthanasia, regardless of the lung problem. Of course, it would be nice to have a living will. If it was clear from this that the patient would prefer to continue living, I would be in favor of Tablet C.” At the same time, this answer suggests that for this vet it does not make a medical difference that the patient is human, as they state that the diagnosis would be sufficient for euthanasia (something the veterinarian is performing regularly with animal patients). Similarly, a veterinarian explains her choice for A in the human coma patient directly referring to her profession: “I am a veterinarian and I make these decisions for my patients every day.”

In the scenarios with the autonomous human patients, there is strong emphasis on human autonomy, e.g., by this veterinarian choosing B for the 70-year-old patient: “Actually, I would like to leave the choice to the patient. It is not for me to decide”, or this control group participant choosing C for the 11-year-old child: “I would only decide this way if he wanted it himself. If he no longer wants to live, I would choose tablet A immediately.” Quite a few statements clearly expressed that any paternalistic choice would be wrong and they would opt for “none of the answers. I would discuss this with Tom and respect his will—no matter what that looks like” (human, 11y, HumMed, C). Some participants explicitly demarcate their decision from those in the other scenarios with non-autonomous animal patients: “In contrast to the dog, Kira can decide for herself. Therefore, all other answers could also be considered” (human, 70y, Control, B). Stressing their communication habits, a human medical professional opting for B in the 70-year-old patient claims: “I talked to Kira. She wanted it that way.” This participant even invented a dialogue with Kira to highlight the self-evidence of asking the patient.

## 4. Discussion

When discussing between-group differences for the decision results, it must be kept in mind that even in cases with significant differences the overall decision patterns in the scenarios are similar for all three groups. Even if, for example, significantly more veterinarians opted for a treatment tablet C in the 70-year-old patient, most participants of the other two groups also chose tablet C. Therefore, the differences between the three groups are addressed subordinately when discussing the overall decision-making patterns and criteria. 

The fracture lines regarding the decisions for one of the four options can be drawn, on the one hand, between older and younger patients. Other things being equal, participants suggest different treatments for the older human and the older canine patient compared to the younger ones. The difference is, however, much more pronounced for the animal than for the human scenarios: Most participants suggest euthanizing the older but treating the younger dog, whereas in all three, for the young and the elderly human patients, most participants considered treatment tablet C the best option. Belonging to the human species, though, does not present a sufficient criterion to be kept alive under all circumstances. For the human coma patient, most participants suggested the euthanasia tablet A. Analyzing these patterns thus allows for the conclusion that species, age and autonomy are intertwined criteria in the decision-making process.

### 4.1. Species

The given circumstances in the scenario present a stark contrast to usual procedures in both human and companion animal medicine in several aspects. 

First, therapies with severe side effects are commonly not applied in companion animal practice even if that means accepting a shortened life of the patient. Chemotherapy is a prominent example. Veterinarians are advised to discontinue chemotherapy if the animal patient shows substantial side effects [8] and overall the most frequently used chemotherapeutic agents are tolerated by a vast majority of the animal patients. Whereas patient owners (potentially including those in our control group) and human medical professionals might not be aware of the specific conditions regarding cancer treatment in veterinary practice [9], veterinarians could be expected to know about those differences. With veterinary oncology being a niche for highly specialized clinicians, though, the knowledge regarding cancer treatment (or other therapies with potential severe side effects) in companion animals might be rather shallow even among veterinarians working in private practices (see e.g., [3]). This contrast between assumptions and actual practice of everyday veterinary medicine might explain the overall high approval of therapeutic tablet C for the young dog even if it would actually never be performed that way. At second glance, the collective support of that option challenges the basic rule in veterinary practice, not to let an animal severely suffer for a moderate time to prolong their life (with a good quality) afterwards. Research on veterinarians’ perspectives and arguments or, in a second step, a public dialogue regarding chances and limitations of the increasing treatment options in veterinary medicine are necessary to additionally shed some light on follow-up questions regarding overtreatment and caregiver burden [10]. 

Second, tablet A obviously represents a choice that is not available in human medicine (in Germany) while describing a standard procedure in veterinary medicine. This difference was, however, picked up remarkably rarely in the free-text answers. Some participants in the animal professional group clearly identified the procedure as euthanasia. In all groups, some participants stated that they would opt for this if they or close family members were in a similar situation, mirroring the ongoing debate on assisted dying. As laid out in the introduction and confirmed by several above-mentioned participant statements, veterinarians’ professional habits can lead to a rather pragmatic and matter-of-fact attitude towards euthanasia that is by definition (and, most likely, supported by the medical discourse) not as acquainted to human medical professionals. Stripped of the usual legal and social constraints and also of the explicit expression “euthanasia” (which is not only uncommon in human medicine but associated with the crimes of the Nazi regime during World War II, in Germany) the fictional text led nevertheless to a considerable proportion of all participant groups administering euthanasia tablet A to a human coma patient. 

Third, continuous palliative sedation as presented in tablet B is not a common practice in veterinary medicine in Germany but is part of the human medical toolbox in EOLD. This is underlined by the fact that participants in the human medical professional group opted for tablet B more often than the other two groups in all but those scenarios involving human children. With the changing role of companion animals who are increasingly perceived as family members, the demand for treatments from animal owners who are familiar with human medicine is growing. That includes procedures at the end-of-life situations. In a recent qualitative study veterinarians report an increasing number of clients asking for alternatives to euthanasia (see [3]) and the hospice and palliative care movement in companion animal medicine is an upcoming branch of veterinary medicine in the USA. What might sound peculiar or at least unfamiliar from a German perspective is already recommended by US veterinarians, stating that “(p)alliative sedation is a reasonable option for patients when clients or veterinarians have moral objections to euthanasia” [11] (p. 40). In none of our participant groups were moral objections the reasons for not euthanizing the dog patient, though. Instead, the main distinguishing reason between tablet A and B was the chance to say goodbye–that is, for the owner rather than for the patient under sedation. Stressing this need to say goodbye (for quite a period of time) again reflects the close, family-like relationship between companion animals and their owners that presents a challenge as well as a chance for veterinarians specialized in geriatrics [12].

Overall, the difference in species seems to have played an implicit role in the decision-making, as both the choices and the explanations in the animal patient scenarios display less ambivalence in the participants’ reasoning. However, the difference is only gradual as argumentative patterns for human and animal patients overlap to a large extent.

### 4.2. Age

A study limitation that is true for all aspects becomes evident for the research focus “age”. The free-text question allowed for exploratory rather than hypothesis-driven research questions making it difficult (a) to quantify and (b) compare argumentation patterns. Apparently, the three introduced dimensions of “age” are represented in those participants’ statements that refer to age, besides the frequent mentioning of “age” lacking further differentiation.

In all three groups, there are participants bringing up the thought that they do not want to put an elderly patient (human or animal) through the six month of stress and suffering because the patient “cannot take it” anymore. In addition, they infer that the younger patient’s body will be able to tolerate the procedure better. Given that, at least in several cases, this is the foremost or the only reason participants mention for their choice, the perspective seems rather reductionist as (a) a patient is generally considered to comprise more than his body and (b) the context of patient owners/family members is excluded in this argument. 

Even more striking, however, are the statements representing the QALY-dimension of age. Given the specifics of commonly tolerated side effects of a veterinary treatment, as elaborated above, the weighing process of the six months with extremely bad quality of life against the (potential) healthy years that follow seems to be a human medical calculation that, in cases like that, would just not be played through in veterinary medicine. Nevertheless, a clear majority of all three groups was willing to put the younger dog through the treatment C procedure as the healing chances were estimated as sufficiently high, and about a fifth of all participants even opted for this in the scenario with the elderly dog. After all, one of the ethical justifications for performing euthanasia in severely ill animals is the basic assumption that, for them, the current suffering outweighs future potential happiness after a time-consuming treatment [13]. An order effect cannot be excluded as a reason for the unexpected results in this regard [14]. It is also possible, that some participants have a general attitude of preserving life under all circumstances and that the killing or letting die of elderly (even animal) patients would be unjustified discrimination, especially in light of the ongoing debate as to whether age should be considered a disease [15,16,17]. 

Against this background, the findings on the fair-innings-dimension were equally surprising. In contrast to the statements of other participants that (in the global North) a 70-year-old person can be expected to have at least ca. 10 more years of life, quite a few participants stated that this patient had lived his life and might as well die peacefully, now. Giving credence to the fact that a huge proportion of all participants stated they would ask the patient or try to learn about their preferences, the minority opinion that a paternalistic decision would be acceptable must be acknowledged. Again, this result can potentially be attributed to the study design, as participants were asked which option they considered to be the best, including a forced decision that intentionally excluded the patient’s perspective. There is the viewpoint, most certainly also a minority opinion, that, to quote one of its most prominent advocates, “living too long is also a loss” [18]. Emanuel is arguing for no longer administering life-prolonging medical interventions, such as treating cancer or pneumonia, after turning 75, a position—without going into the details of his argumentation—that can be subsumed under the fair innings dimension. Possibly, those study participants judging the human patient to have had a full, complete or rich life share the thought that nothing that would come after the offered treatment would make the patient’s life as a whole better. Transferring this argument to nonhuman animals, though, includes several presuppositions that are not elaborated in the free-text answers, some of which are closely related to general considerations regarding animal euthanasia. Assuming that participants agreed that animals do not share the concept of a “life span” or have expectations regarding their life as a whole, could animals nonetheless reach a time in life above which living would be a loss? What criteria are used to determine a “full life span” for an animal? To what extent do animals suffer from being old, i.e., in a different and, if arguing with Emanuel, less valuable state of themselves? It can be doubted that, arguing with Emanuel’s criteria, it is purposeful to talk about an animal “having lived their life”. However, the study participants may have considered further criteria that are more applicable to animals (such as having lived through different essential stages of life or having experienced aspects integral to an animal’s life) to support their statements.

Finally, despite comparable decision patterns (see Figure 1 and Figure 5), age represents an apparent difference between the elderly dog and the coma patient, who share morally relevant properties otherwise: Both are, in a narrow sense, not autonomous and cannot clearly express their preferences. Arguing from the fair-innings-dimension, though, the dog has lived almost a full “Labrador Retriever’s life span”, whereas most people would agree that the 30-year-old coma patient will die earlier than she or he could have expected. In this case, the QUALY-dimension of age seems to be more relevant for the decision-making. As both have no reason to further expect a long life with a high quality of life, a majority of the participants votes for ending their life with tablet A or B. 

### 4.3. Autonomy

Autonomy can be understood as a graded property. With different accounts of autonomy described in the literature [19,20,21] and the additional debate on animal agency and autonomy [22], the in-depth analysis of the relationships between the different understandings of autonomy in the scenarios and their potential relevance as uttered in the free-text statements would go beyond the scope of this article. Rather, autonomy will be understood in a negative definition as described in the introduction, closely linked to the capacity to give consent. (Not)/being autonomous in the here and now sense refers to shared properties of the so-called marginal cases and nonhuman animals, close to the five necessary conditions for capacity to consent as defined by Jox [23], i.e., a patient’s ability to

1. understand decision-relevant facts, consequences and risks of, e.g., a treatment
2. apply the relevant information to their own situation
3. weigh arguments for and against different options in the light of their own values and attitudes
4. make a decision based on that weighing process
5. communicate that decision.

Kira and Tom, as an infant, comatose or dog patient, undoubtedly do not fulfill those requirements. That is why in those cases the necessity to make a decision for them is more obvious than in case of the 11-year-old child or the 70-year-old adult. 

The hesitation when being forced to decide for these two patients is displayed in several evasive participant answers who would have liked to include the patient’s will, too, independent of their choices for Tablets A, B, or C. Several statements indicated a cascade from talking directly to the patient, looking for an advance directive, to asking family and friends in order to respect the patient’s autonomy. Interestingly, despite the clear difference in (current) autonomy, the decision-making for the infant and the 11-year-old was almost exactly the same. Here, the combination of a long life expectancy and the chances of being healed were judged to be most relevant.

Dealing with non-autonomous patients presented fewer difficulties for the participants according to the free-text answers. The paternalistic act of deciding for tablet A or B was, in line with Jox’s above-mentioned criteria, frequently justified by the incapability of the animal to understand the purpose of a therapy (tablet C) or to express their current level of suffering (which is occasionally underestimated even by companion animal owners). Arguably, this challenge could equally be attributed to a human deficit in precisely perceiving the animals’ suffering, rather than to the animals’ deficit in communicating their physical and mental state. Several times, respect was paid to the (autonomous) animal owner’s preferences when choosing tablet B to say goodbye rather than to the presumed preferences of the animal patient. In the non-autonomous coma patient, participants did not mention autonomy as an influential factor, in contrast to the patient’s prospective quality of life (see above). 

Methodologically speaking, the obtained data support the thought experiment’s success. Participants were able and willing to distance themselves from constraints and frameworks they experience in their everyday life and to get involved in the scenario. Intentionally, the authors did not ask the participants for a personal choice if they were in the patient’s, the family’s, or advising physician’s situation but for their judgement on the best option in the case as an observer. However, the very vague wording, “Which option do you consider to be right?” left room for the participants to pick a role, either as patient, family member, or, especially in case of the human medical and veterinary professionals, as treating doctors. The spectrum of answers underlines the process some participants have gone through; first deciding on a role or perspective (“If it was me/my father…”, “we (i.e., veterinarians) treat such cases…”, “I would want that for my dog” etc.) and then on one of the four options.

This was also the aspect most clearly distinguishing professional and private experiences of the three study groups, underlining the slight differences in the answering patterns. Veterinarians who regularly euthanize patients referred to that habit and corresponding justifications whereas human medical professionals, being used to palliative treatment options, elaborated on that decision in a professional, habitual way, and the control group rather fell back on examples from their private life.

At the same time, despite the careful phrasing, the question might have suggested that there is, objectively, a “right” choice, leading the participants to pick and mention potential objective criteria for supporting their choices. To be more thorough, a further question in the study might have pointed out more clearly to what extent participants are convinced that there is a right answer (or, rather, several justifiable opinions) in this matter. A further obstacle in the free-text analysis occurred when participants kept referring to their former answers without being precise (“Like in the other scenario”, “same reason”, “see above”, etc.). With the randomized order of the scenarios, the analysis of those statements would have been at least very cumbersome if not speculative. 

Another phenomenon worth discussing is the non-completion rate of almost 40%. Even though there is no consensus in the literature on what percentage constitutes an acceptable completion rate in web surveys [24], the authors (as well as an anonymous reviewer) agree that 40% is a number of potential participants worth commenting on. Besides well-known factors that can negatively affect completion rates in general (e.g., length of the survey, number of questions, forced responses, display style, incentives, survey fatigue, depth of thought required, etc. [24,25,26]), certain specifics of this survey may have further diminished completion rates. Most notably, the described scenarios did not differ from one another except for patient characteristics.

Some participants, expecting to be exposed to a wider range of end-of-life situations, might have been “disappointed” relatively quickly. Others may have felt uneasy or even exposed when learning that their moral intuitions to treat certain patients differently or similarly might be incompatible with their ability to give moral reasons to do so in their free-text answers. It is also possible that respondents might have evaluated the described patients or situations to be not so different from one another (e.g., because they assess that in all cases one should simply ask the patient or the patient’s family for their preferences or because they think that it is always worth trying to save a patient’s life regardless of the chance of success) so that they simply lost patience. All these experiences may have led participants to discontinue the survey at some point. Of course, these possibilities are mere speculations—nonetheless it may be promising to research these hypothetical conjectures in the future.

In a follow-up study, instead of asking for free-text explanation a list of factors extracted from the exploratory results discussed here could be displayed to the study participants in order to select those that most influenced their choices. Alternatively, a qualitative interview study with participants from all three groups could more thoroughly shed light on their reasoning and explanations for their decisions.

## 5. Conclusions

This study did not present different established professional attitudes and practices regarding EOLD in veterinary and human practice. That kind of research has been done before (e.g., [27,28]) and may have led to certain expectations regarding fundamental differences in the attitudes of veterinarians and human medical professionals. 

This thought experiment, however, succeeded in crossing disciplinary boundaries and, thereby, shedding light on more fundamental criteria of decision-making. With the difference in species being the most obvious between the scenarios and at the same time only a minor factor when making and reasoning about their decisions, the participants impressively demonstrated that there are more fine-grained criteria for both groups of patients, despite professional habits and familiarity with legal constraints. 

Besides the consistently mentioned criterion “suffering” that the participants ascribed to all patients and that they made the most basic reason for their decisions, the overarching criteria “age” and “autonomy”, in the above-described sense, explained most decision-patterns with “species” being a subordinate criterion, leading to a stronger tendency to kill the patient in case of doubt. 

The interesting findings that do not mirror the participants’ experiences in their actual life, i.e., palliative sedation of a dog, the aggressive treatment with long-lasting side effects for an elderly dog, euthanasia or aggressive treatment of a human coma patient, present cases of some disagreement in decision-making between the groups. This points towards interesting opportunities for both, human medical and veterinary medicine, to intensify their inter-professional dialogue on convergences and divergences and, based on that, to critically analyze their ethical reasoning and decision-making processes, and, potentially, to learn from the interdisciplinary exchange, that will in turn influence public policy making.

## Data Availability

The data presented in this study are available on request from the corresponding author. The data are not publicly available due to protection of the participants’ anonymity.

## Supplementary Materials

The following supporting information can be downloaded at: https://www.mdpi.com/article/10.3390/ani12192494/s1, Material S1: Survey_German; Material S2: Data Protection.
