# Supplementary material for: Killing Kira, Letting Tom Go?—An Empirical Study on Intuitions Regarding End-of-Life Decisions in Companion Animals and Humans
Source: Animals (Basel). 2022 Sep 20;12(19):2494. doi: 10.3390/ani12192494 (PMC9559485; doi:10.3390/ani12192494)
Supplement: Supplementary file 1 [file animals-12-02494-s001.zip › S1.pdf]

Lebensende Tier und Mensch

Liebe*r Teilnehmer*in,

wir möchten Sie einladen, an einem Gedankenexperiment teilzunehmen: Im Folgenden werden Ihnen 6 unterschiedliche fiktive Szenarien vorgestellt. Bitte lesen Sie sich die Informationen und die Frage zunächst genau durch und beantworten im Anschluss die jeweilige Frage. Es gibt keine objektiv richtige und falsche Antwort. Auch sollten Sie davon ausgehen, dass alle Antwortoptionen möglich (also z.B. nicht illegal oder technisch ausgeschlossen) sind. Da dies ein Gedankenexperiment ist, geht es nur um Ihre eigene, persönliche Einschätzung, was das Beste wäre. Sie können sich in dem Fall mit niemandem absprechen. Sie müssen nicht die Frage einer möglichen Zustimmung/Einwilligung (durch wen auch immer) klären. Wenn Sie aufgefordert werden, freie Antworten zu formulieren, genügen Stichworte.

In jedem der folgenden 6 Szenarien wird Ihnen ein ähnliches medizinisches Setting begegnen, die Patient*innen sind aber verschiedene (Menschen und andere Tiere, Ältere und Jüngere, unterschiedliche Lebensumstände).

In dieser Umfrage sind 28 Fragen enthalten.

Zuordnung zur Fragegruppe

```
1 {if(randnumber > 0, randnumber, rand(1,2))}
```

Fragegruppe 1

2

Tom ist ein **11-jähriger Labradorrüde.** Bei ihm wurde eine fortschreitende Lungenkrankheit diagnostiziert und sein aktueller Zustand ist schlecht. Er hat Schwierigkeiten bei der Atmung, frisst nicht mehr und hat kaum noch Interesse an Alltagsaktivitäten wie Spaziergängen.

Es stehen folgende Optionen zur Auswahl:

- Wird kein Medikament verabreicht, wird Tom an Luftnot leiden und in kurzer Zeit ersticken.
- Wird Tablette A verabreicht, wird Tom unmittelbar schmerz- und angstfrei einschlafen und versterben.
- Wird Tablette B verabreicht, wird Tom ruhig gestellt, sodass er weder Angst noch Schmerzen oder Unwohlsein verspürt. Er wird voraussichtlich innerhalb der nächsten zwei Wochen an seiner Krankheit versterben.
- Wird Tablette C verabreicht, wird Tom etwa sechs Monate lang starke Nebenwirkungen wie Übelkeit, Appetitlosigkeit, Schwindel, starke Müdigkeit und Antriebslosigkeit verspüren. Danach besteht eine 70%ige Chance, dass die Lunge geheilt ist. Mit 30%iger Wahrscheinlichkeit kann das Lungenversagen auf diese Weise nicht aufgehalten werden.

Welche Option halten Sie für richtig?

*

❗ Bitte wählen Sie eine der folgenden Antworten:
Bitte wählen Sie nur eine der folgenden Antworten aus:

◯ kein Medikament verabreichen
◯ Tablette A verabreichen
◯ Tablette B verabreichen
◯ Tablette C verabreichen

3 Ich habe diese Auswahl getroffen, weil... *

Bitte geben Sie Ihre Antwort hier ein:

Fragegruppe 3

4

Tom ist ein **70 Jahre alter Mann.** Bei ihm wurde eine fortschreitende Lungenkrankheit diagnostiziert und sein aktueller Zustand ist schlecht. Er hat Schwierigkeiten bei der Atmung, isst nicht mehr und hat kaum noch Interesse an Alltagsaktivitäten wie Spaziergängen.

Es stehen folgende Optionen zur Auswahl:

- Wird kein Medikament verabreicht, wird Tom an Luftnot leiden und in kurzer Zeit ersticken.
- Wird Tablette A verabreicht, wird Tom unmittelbar schmerz- und angstfrei einschlafen und versterben.
- Wird Tablette B verabreicht, wird Tom ruhig gestellt, sodass er weder Angst noch Schmerzen oder Unwohlsein verspürt. Er wird voraussichtlich innerhalb der nächsten zwei Wochen an seiner Krankheit versterben.
- Wird Tablette C verabreicht, wird Tom etwa sechs Monate lang starke Nebenwirkungen wie Übelkeit, Appetitlosigkeit, Schwindel, starke Müdigkeit und Antriebslosigkeit verspüren. Danach besteht eine 70%ige Chance, dass die Lunge geheilt ist. Mit 30%iger Wahrscheinlichkeit kann das Lungenversagen auf diese Weise nicht aufgehalten werden.

Welche Option halten Sie für richtig?

*

❗ Bitte wählen Sie eine der folgenden Antworten:
Bitte wählen Sie nur eine der folgenden Antworten aus:

◯ kein Medikament verabreichen

◯ Tablette A verabreichen

◯ Tablette B verabreichen

◯ Tablette C verabreichen

5 Ich habe diese Auswahl getroffen, weil... *

Bitte geben Sie Ihre Antwort hier ein:

Fragegruppe 5

6

Tom ist ein **30-jähriger Mann**, der seit 5 Jahren im **irreversiblen Koma** liegt. Bei ihm wurde nun eine fortschreitende Lungenkrankheit diagnostiziert und sein aktueller Zustand ist schlecht. Er hat Schwierigkeiten bei der Atmung und zeigt messbare Anzeichen von Stress.

Es stehen folgende Optionen zur Auswahl:

- Wird kein Medikament verabreicht, wird Tom an Luftnot leiden und in kurzer Zeit ersticken.
- Wird Tablette A verabreicht, wird Tom unmittelbar schmerz- und angstfrei einschlafen und versterben.
- Wird Tablette B verabreicht, wird Tom ruhig gestellt, sodass er weder Angst noch Schmerzen oder Unwohlsein verspürt. Er wird voraussichtlich innerhalb der nächsten zwei Wochen an seiner Krankheit versterben.
- Wird Tablette C verabreicht, besteht eine 70%ige Chance, dass die Lunge nach 6 Monaten geheilt ist. Die üblichen Nebenwirkungen wie Übelkeit, Schwindel und starke Müdigkeit wird Tom nicht spüren. Mit 30%iger Wahrscheinlichkeit kann das Lungenversagen auf diese Weise nicht aufgehalten werden.

Welche Option halten Sie für richtig?

*

❗ Bitte wählen Sie eine der folgenden Antworten:
Bitte wählen Sie nur eine der folgenden Antworten aus:

◯ kein Medikament verabreichen

◯ Tablette A verabreichen

◯ Tablette B verabreichen

◯ Tablette C verabreichen

7 Ich habe diese Auswahl getroffen, weil... *

Bitte geben Sie Ihre Antwort hier ein:

Fragegruppe 4

8

Tom ist ein 6 Monate altes Baby. Bei ihm wurde eine fortschreitende Lungenkrankheit diagnostiziert und sein aktueller Zustand ist schlecht. Er hat Schwierigkeiten bei der Atmung, trinkt nicht mehr und hat kaum noch Interesse am Kontakt mit seiner Umwelt.

Es stehen folgende Optionen zur Auswahl:

- Wird kein Medikament verabreicht, wird Tom an Luftnot leiden und in kurzer Zeit ersticken.
- Wird Tablette A verabreicht, wird Tom unmittelbar schmerz- und angstfrei einschlafen und versterben.
- Wird Tablette B verabreicht, wird Tom ruhig gestellt, sodass er weder Angst noch Schmerzen oder Unwohlsein verspürt. Er wird voraussichtlich innerhalb der nächsten zwei Wochen an seiner Krankheit versterben.
- Wird Tablette C verabreicht, wird Tom etwa sechs Monate lang starke Nebenwirkungen wie Übelkeit, Appetitlosigkeit, Schwindel, starke Müdigkeit und Antriebslosigkeit verspüren. Danach besteht eine 70%ige Chance, dass die Lunge geheilt ist. Mit 30%iger Wahrscheinlichkeit kann das Lungenversagen auf diese Weise nicht aufgehalten werden.

Welche Option halten Sie für richtig?

*

❗ Bitte wählen Sie eine der folgenden Antworten:
Bitte wählen Sie nur eine der folgenden Antworten aus:

◯ kein Medikament verabreichen

◯ Tablette A verabreichen

◯ Tablette B verabreichen

◯ Tablette C verabreichen

9 Ich habe diese Auswahl getroffen, weil... *

Bitte geben Sie Ihre Antwort hier ein:

Fragegruppe 6

10

Tom ist ein **2-jähriger Labradorrüde.** Bei ihm wurde eine fortschreitende Lungenkrankheit diagnostiziert und sein aktueller Zustand ist schlecht. Er hat Schwierigkeiten bei der Atmung, frisst nicht mehr und hat kaum noch Interesse an Alltagsaktivitäten wie Spaziergängen.

Es stehen folgende Optionen zur Auswahl:

- Wird kein Medikament verabreicht, wird Tom an Luftnot leiden und in kurzer Zeit ersticken.
- Wird Tablette A verabreicht, wird Tom unmittelbar schmerz- und angstfrei einschlafen und versterben.
- Wird Tablette B verabreicht, wird Tom ruhig gestellt, sodass er weder Angst noch Schmerzen oder Unwohlsein verspürt. Er wird voraussichtlich innerhalb der nächsten zwei Wochen an seiner Krankheit versterben.
- Wird Tablette C verabreicht, wird Tom etwa sechs Monate lang starke Nebenwirkungen wie Übelkeit, Appetitlosigkeit, Schwindel, starke Müdigkeit und Antriebslosigkeit verspüren. Danach besteht eine 70%ige Chance, dass die Lunge geheilt ist. Mit 30%iger Wahrscheinlichkeit kann das Lungenversagen auf diese Weise nicht aufgehalten werden.

Welche Option halten Sie für die richtige?

*

❗ Bitte wählen Sie eine der folgenden Antworten:
Bitte wählen Sie nur eine der folgenden Antworten aus:

- ◯ kein Medikament verabreichen
- ◯ Tablette A verabreichen
- ◯ Tablette B verabreichen
- ◯ Tablette C verabreichen

11 Ich habe diese Auswahl getroffen, weil... *

Bitte geben Sie Ihre Antwort hier ein:

Fragegruppe 7

12

Tom ist ein **11 Jahre alter Junge.** Bei ihm wurde eine fortschreitende Lungenkrankheit diagnostiziert und sein aktueller Zustand ist schlecht. Er hat Schwierigkeiten bei der Atmung, isst nicht mehr und hat kaum noch Interesse an Alltagsaktivitäten.

Es stehen folgende Optionen zur Auswahl:

- Wird kein Medikament verabreicht, wird Tom an Luftnot leiden und in kurzer Zeit ersticken.
- Wird Tablette A verabreicht, wird Tom unmittelbar schmerz- und angstfrei einschlafen und versterben.
- Wird Tablette B verabreicht, wird Tom ruhig gestellt, sodass er weder Angst noch Schmerzen oder Unwohlsein verspürt. Er wird voraussichtlich innerhalb der nächsten zwei Wochen an seiner Krankheit versterben.
- Wird Tablette C verabreicht, wird Tom etwa sechs Monate lang starke Nebenwirkungen wie Übelkeit, Appetitlosigkeit, Schwindel, starke Müdigkeit und Antriebslosigkeit verspüren. Danach besteht eine 70%ige Chance, dass die Lunge geheilt ist. Mit 30%iger Wahrscheinlichkeit kann das Lungenversagen auf diese Weise nicht aufgehalten werden.

Welche Option halten Sie für richtig?

*

❗ Bitte wählen Sie eine der folgenden Antworten:
Bitte wählen Sie nur eine der folgenden Antworten aus:

◯ kein Medikament verabreichen

◯ Tablette A verabreichen

◯ Tablette B verabreichen

◯ Tablette C verabreichen

13 Ich habe diese Auswahl getroffen, weil... *

Bitte geben Sie Ihre Antwort hier ein:

Fragegruppe 1a

14

Kira ist eine **11-jährige Labradorhündin.** Bei ihr wurde eine fortschreitende Lungenkrankheit diagnostiziert und ihr aktueller Zustand ist schlecht. Sie hat Schwierigkeiten bei der Atmung, frisst nicht mehr und hat kaum noch Interesse an Alltagsaktivitäten wie Spaziergängen.

Es stehen folgende Optionen zur Auswahl:

- Wird kein Medikament verabreicht, wird Kira an Luftnot leiden und in kurzer Zeit ersticken.
- Wird Tablette A verabreicht, wird Kira unmittelbar schmerz- und angstfrei einschlafen und versterben.
- Wird Tablette B verabreicht, wird Kira ruhig gestellt, sodass sie weder Angst noch Schmerzen oder Unwohlsein verspürt. Sie wird voraussichtlich innerhalb der nächsten zwei Wochen an ihrer Krankheit versterben.
- Wird Tablette C verabreicht, wird Kira etwa sechs Monate lang starke Nebenwirkungen wie Übelkeit, Appetitlosigkeit, Schwindel, starke Müdigkeit und Antriebslosigkeit verspüren. Danach besteht eine 70%ige Chance, dass die Lunge geheilt ist. Mit 30%iger Wahrscheinlichkeit kann das Lungenversagen auf diese Weise nicht aufgehalten werden.

Welche Option halten Sie für richtig?

*

❗ Bitte wählen Sie eine der folgenden Antworten:
Bitte wählen Sie nur eine der folgenden Antworten aus:

◯ kein Medikament verabreichen

◯ Tablette A verabreichen

◯ Tablette B verabreichen

◯ Tablette C verabreichen

15 Ich habe diese Auswahl getroffe, weil... *

Bitte geben Sie Ihre Antwort hier ein:

Fragebruppe 2a

16

Kira ist eine **70 Jahre alte Frau.** Bei ihr wurde eine fortschreitende Lungenkrankheit diagnostiziert und ihr aktueller Zustand ist schlecht. Sie hat Schwierigkeiten bei der Atmung, isst nicht mehr und hat kaum noch Interesse an Alltagsaktivitäten wie Spaziergängen.

Es stehen folgende Optionen zur Auswahl:

- Wird kein Medikament verabreicht, wird Kira an Luftnot leiden und in kurzer Zeit ersticken.
- Wird Tablette A verabreicht, wird Kira unmittelbar schmerz- und angstfrei einschlafen und versterben.
- Wird Tablette B verabreicht, wird Kira ruhig gestellt, sodass sie weder Angst noch Schmerzen oder Unwohlsein verspürt. Sie wird voraussichtlich innerhalb der nächsten zwei Wochen an ihrer Krankheit versterben.
- Wird Tablette C verabreicht, wird Kira etwa sechs Monate lang starke Nebenwirkungen wie Übelkeit, Appetitlosigkeit, Schwindel, starke Müdigkeit und Antriebslosigkeit verspüren. Danach besteht eine 70%ige Chance, dass die Lunge geheilt ist. Mit 30%iger Wahrscheinlichkeit kann das Lungenversagen auf diese Weise nicht aufgehalten werden.

Welche Option halten Sie für richtig?

*

❗ Bitte wählen Sie eine der folgenden Antworten:
Bitte wählen Sie nur eine der folgenden Antworten aus:

◯ kein Medikament verabreichen

◯ Tablette A verabreichen

◯ Tablette B verabreichen

◯ Tablette C verabreichen

17 Ich habe diese Auswahl getroffen, weil... *

Bitte geben Sie Ihre Antwort hier ein:

Fragegruppe 3a

18

Kira ist ein **6 Monate altes Baby.** Bei ihr wurde eine fortschreitende Lungenkrankheit diagnostiziert und ihr aktueller Zustand ist schlecht. Sie hat Schwierigkeiten bei der Atmung, trinkt nicht mehr und hat kaum noch Interesse am Kontakt mit ihrer Umwelt.

Es stehen folgende Optionen zur Auswahl:

- Wird kein Medikament verabreicht, wird Kira an Luftnot leiden und in kurzer Zeit ersticken.
- Wird Tablette A verabreicht, wird Kira unmittelbar schmerz- und angstfrei einschlafen und versterben.
- Wird Tablette B verabreicht, wird Kira ruhig gestellt, sodass sie weder Angst noch Schmerzen oder Unwohlsein verspürt. Sie wird voraussichtlich innerhalb der nächsten zwei Wochen an ihrer Krankheit versterben.
- Wird Tablette C verabreicht, wird Kira etwa sechs Monate lang starke Nebenwirkungen wie Übelkeit, Appetitlosigkeit, Schwindel, starke Müdigkeit und Antriebslosigkeit verspüren. Danach besteht eine 70%ige Chance, dass die Lunge geheilt ist. Mit 30%iger Wahrscheinlichkeit kann das Lungenversagen auf diese Weise nicht aufgehalten werden.

Welche Option halten Sie für richtig?

*

❗ Bitte wählen Sie eine der folgenden Antworten:
Bitte wählen Sie nur eine der folgenden Antworten aus:

◯ kein Medikament verabreichen

◯ Tablette A verabreichen

◯ Tablette B verabreichen

◯ Tablette C verabreichen

19 Ich habe diese Auswahl getroffen, weil... *

Bitte geben Sie Ihre Antwort hier ein:

Fragegruppe4a

20

Kira ist eine **30-jährige Frau**, die seit 5 Jahren im **irreversiblen Koma** liegt. Bei ihr wurde nun eine fortschreitende Lungenkrankheit diagnostiziert und ihr aktueller Zustand ist schlecht. Sie hat Schwierigkeiten bei der Atmung und zeigt messbare Anzeichen von Stress.

Es stehen folgende Optionen zur Auswahl:

- Wird kein Medikament verabreicht, wird Kira an Luftnot leiden und in kurzer Zeit ersticken.
- Wird Tablette A verabreicht, wird Kira unmittelbar schmerz- und angstfrei versterben.
- Wird Tablette B verabreicht, wird Kira ruhig gestellt, sodass sie weder Angst noch Schmerzen oder Unwohlsein verspürt. Sie wird voraussichtlich innerhalb der nächsten zwei Wochen an seiner Krankheit versterben.
- Wird Tablette C verabreicht, besteht eine 70%ige Chance, dass die Lunge nach 6 Monaten geheilt ist. Die üblichen Nebenwirkungen wie Übelkeit, Schwindel und starke Müdigkeit wird Kira nicht spüren. Mit 30%iger Wahrscheinlichkeit kann das Lungenversagen auf diese Weise nicht aufgehalten werden.

Welche Option halten Sie für richtig?

*

❗ Bitte wählen Sie eine der folgenden Antworten:
Bitte wählen Sie nur eine der folgenden Antworten aus:

◯ kein Medikament verabreichen

◯ Tablette A verabreichen

◯ Tablette B verabreichen

◯ Tablette C verabreichen

21 Ich habe diese Auswahl getroffen, weil... *

Bitte geben Sie Ihre Antwort hier ein:

Fragegruppe 5a

22

Kira ist eine **2-jährige Labradorhündin.** Bei ihr wurde eine fortschreitende Lungenkrankheit diagnostiziert und ihr aktueller Zustand ist schlecht. Sie hat Schwierigkeiten bei der Atmung, frisst nicht mehr und hat kaum noch Interesse an Alltagsaktivitäten wie Spaziergängen.

Es stehen folgende Optionen zur Auswahl:

- Wird kein Medikament verabreicht, wird Kira an Luftnot leiden und in kurzer Zeit ersticken.
- Wird Tablette A verabreicht, wird Kira unmittelbar schmerz- und angstfrei einschlafen und versterben.
- Wird Tablette B verabreicht, wird Kira ruhig gestellt, sodass sie weder Angst noch Schmerzen oder Unwohlsein verspürt. Sie wird voraussichtlich innerhalb der nächsten zwei Wochen an ihrer Krankheit versterben.
- Wird Tablette C verabreicht, wird Kira etwa sechs Monate lang starke Nebenwirkungen wie Übelkeit, Appetitlosigkeit, Schwindel, starke Müdigkeit und Antriebslosigkeit verspüren. Danach besteht eine 70%ige Chance, dass die Lunge geheilt ist. Mit 30%iger Wahrscheinlichkeit kann das Lungenversagen auf diese Weise nicht aufgehalten werden.

Welche Option halten Sie für die richtige?

*

❗ Bitte wählen Sie eine der folgenden Antworten:
Bitte wählen Sie nur eine der folgenden Antworten aus:

◯ kein Medikament verabreichen

◯ Tablette A verabreichen

◯ Tablette B verabreichen

◯ Tablette C verabreichen

23 Ich habe diese Auswahl getroffen, weil... *

Bitte geben Sie Ihre Antwort hier ein:

Frageruppe6a

24

Kira ist ein **11 Jahre altes Mädchen.** Bei ihr wurde eine fortschreitende Lungenkrankheit diagnostiziert und ihr aktueller Zustand ist schlecht. Sie hat Schwierigkeiten bei der Atmung, isst nicht mehr und hat kaum noch Interesse an Alltagsaktivitäten.

Es stehen folgende Optionen zur Auswahl:

- Wird kein Medikament verabreicht, wird Kira an Luftnot leiden und in kurzer Zeit ersticken.
- Wird Tablette A verabreicht, wird Kira unmittelbar schmerz- und angstfrei einschlafen und versterben.
- Wird Tablette B verabreicht, wird Kira ruhig gestellt, sodass sie weder Angst noch Schmerzen oder Unwohlsein verspürt. Sie wird voraussichtlich innerhalb der nächsten zwei Wochen an ihrer Krankheit versterben.
- Wird Tablette C verabreicht, wird Kira etwa sechs Monate lang starke Nebenwirkungen wie Übelkeit, Appetitlosigkeit, Schwindel, starke Müdigkeit und Antriebslosigkeit verspüren. Danach besteht eine 70%ige Chance, dass die Lunge geheilt ist. Mit 30%iger Wahrscheinlichkeit kann das Lungenversagen auf diese Weise nicht aufgehalten werden.

Welche Option halten Sie für richtig?

*

❶ Bitte wählen Sie eine der folgenden Antworten:
Bitte wählen Sie nur eine der folgenden Antworten aus:

◯ kein Medikament verabreichen
◯ Tablette A verabreichen
◯ Tablette B verabreichen
◯ Tablette C verabreichen

25 Ich habe diese Auswahl getroffen, weil... *

Bitte geben Sie Ihre Antwort hier ein:

Fragegruppe 2

Demografische Daten

26

Bitte machen Sie nun einige Angaben zu Ihrer Person.

Sie sind...

*

❗ Bitte wählen Sie eine der folgenden Antworten:
Bitte wählen Sie nur eine der folgenden Antworten aus:

- ◯ ...im Bereich der Humanmedizin tätig
- ◯ ...im Bereich der Tiermedizin tätig
- ◯ Sonstiges

27 Inwiefern haben Sie beruflich mit Lebensende-Situationen zu tun? *

Bitte geben Sie Ihre Antwort hier ein:

28 Haben Sie sonstige Anmerkungen zu dieser Studie?

Bitte geben Sie Ihre Antwort hier ein:

Sie haben nun alle Fragen bearbeitet.

Besten Dank für Ihre Teilnahme!

25.08.2021 – 11:43

Übermittlung Ihres ausgefüllten Fragebogens:
Vielen Dank für die Beantwortung des Fragebogens.
