# Supplementary material for: Killing Kira, Letting Tom Go?—An Empirical Study on Intuitions Regarding End-of-Life Decisions in Companion Animals and Humans
Source: Animals (Basel). 2022 Sep 20;12(19):2494. doi: 10.3390/ani12192494 (PMC9559485; doi:10.3390/ani12192494)
Supplement: Supplementary file 1 [file animals-12-02494-s001.zip › S2.pdf]

Datenschutzhinweise und Einwilligungserklärung

1. Zweck der Verarbeitung
Wir erheben und verarbeiten die personenbezogenen Daten zur Teilnahme an der oben genannten Umfrage. Zweck dieser Umfrage ist eine Untersuchung der Einstellungen zu Lebensendesituationen bei Menschen und Heimtieren. Die Ergebnisse der Umfrage werden durch die Studienverantwortlichen zu Forschungs-, Vortrags-, und Publikationszwecken genutzt; sollten personenbeziehbare Informationen vorliegen, werden diese zuvor anonymisiert, Art. 6 I 1 lit. e i.V.m. 89 DSGVO, § 3 I 1 Nr. 1 NHG, § 13 NDSG.

2. Rechtsgrundlage der Verarbeitung
Die Teilnahme an der Umfrage erfolgt freiwillig. Beim Ausfüllen der Umfrage wird Ihre IP-Adresse an den Klinik-Server übermittelt und dort – zumindest vorübergehend – gespeichert. Durch die Teilnahme an der Umfrage willigen Sie in diese Speicherung gem. Art. 6 I 1 lit. a DSGVO ein. Weitere personenbezogene Daten werden nicht verarbeitet. Alle Daten werden sicher und vertraulich behandelt. Die Umfrage wurde mit Hilfe einer hierfür vorgesehenen Software von LimeSurvey auf dem Klinik- Server entworfen. Darüber hinaus können Datenschutzhinweise von LimeSurvey online eingesehen werden: https://www.limesurvey.org/de/richtlinien/datenschutzrichtlinie. Die Möglichkeit der Nutzung zu Lehr-, Forschungs-, Vortrags- und Publikationszwecken ergibt sich aus Art. 6 I 1 lit. e i.V.m. § 13 NDSG. Die Möglichkeit zur Datenverarbeitung zu Evaluationszwecken lässt sich aus Art. 6 I 1 lit. e i.V.m. § 5 NHG ableiten.

3. Dauer der Verarbeitung
Die personenbezogenen Daten werden für die Dauer der Umfrage und des darüberhinausgehenden Forschungsvorhabens gespeichert. Eine Löschung erfolgt automatisch, spätestens 6 Monate nach Beendigung des Vorhabens. Gesetzliche Speicherpflichten bleiben davon unberührt.

4. Rechte der Betroffenen
Sie haben gegenüber der unten genannten Institution ein Recht auf Auskunft über die Sie betreffenden personenbezogenen Daten sowie gegebenenfalls auf Berichtigung, Löschung oder auf Einschränkung der Verarbeitung dieser Daten und ein Widerspruchsrecht gegen die Verarbeitung sowie ein etwaiges Recht auf Datenübertragbarkeit. Ihre etwaige Einwilligung können Sie jederzeit mit Wirkung für die Zukunft widerrufen, Art. 7 III DSGVO Zudem haben Sie ein Recht auf Beschwerde bei der Aufsichtsbehörde, wenn Sie der Ansicht sind, dass die Verarbeitung der Sie betreffenden personenbezogenen Daten gegen die Rechtsvorschriften verstößt. Die zuständige Aufsichtsbehörde ist die Landesbeauftragte für den Datenschutz Niedersachsen.

Projektleitung

Prof. Dr. Peter Kunzmann

AG Angewandte Ethik in der Tiermedizin

Stiftung Tierärztliche Hochschule Hannover

Institut für Tierhygiene, Tierschutz und Nutztierethologie

Bischofsholer Damm 15 (Gebäude 116)

30173 Hannover

Tel.: +49 511 856-8956

E-Mail: Peter.Kunzmann@tiho-hannover.de

Ansprechpartnerin

Kirsten Persson

AG Angewandte Ethik in der Tiermedizin

Stiftung Tierärztliche Hochschule Hannover

Institut für Tierhygiene, Tierschutz und Nutztierethologie

Bischofsholer Damm 15 (Gebäude 116)

30173 Hannover

Email: Kirsten.Persson@tiho-hannover.de

Datenschutzbeauftragter Stiftung Tierärztliche Hochschule

Wolfgang Rottwinkel
Bünteweg 2, 30559 Hannover
Tel.: 0511 953 8015
Fax: 0511 953 828015
E-Mail: datenschutz@tihohannover.de
